# Towards a Multidimensional Model of Neurocognitive Disorders (MOND Model): Integrating Evidence from a Critical Review into a Model for Future Research

**DOI:** 10.3390/jpm16070363

**Published:** 2026-07-03

**Authors:** Joana O. Pinto, Bruno Peixoto, Artemisa R. Dores, Fernando Barbosa

**Affiliations:** 1 Department of Social and Behavioural Sciences, University Institute of Health Sciences, CESPU, 4585-116 Gandra, Portugal; bruno.peixoto@iucs.cespu.pt; 2 1H-TOXRUN—One Health Toxicology Research Unit, University Institute of Health Sciences, CESPU, 4585-116 Gandra, Portugal; 3 Translational Toxicology Research Laboratory, UCIBIO—Applied Molecular Biosciences Unit, University Institute of Health Sciences, CESPU, 4585-116 Gandra, Portugal; 4 Laboratory of Neuropsychophysiology, Faculty of Psychology and Education Sciences, University of Porto, 4200-135 Porto, Portugal; 5 Associate Laboratory i4HB—Institute for Health and Bioeconomy, University Institute of Health Science, CESPU, 4585-116 Gandra, Portugal; 6 CIR, ESS, Polytechnic of Porto, Rua Dr. António Bernardino de Almeida nº 400, 4200-072 Porto, Portugal

**Keywords:** MOND model, neurocognitive disorders, classification, diagnosis, prognosis

## Abstract

The main purpose of this work is to critically review the literature on neurocognitive disorders (ND) diagnosis. A critical review was conducted in PubMed, Scopus, and EBSCO. Systematic reviews and meta-analyses focusing on ND diagnosis were included. The selected studies were critically analyzed and conceptually integrated to identify relevant dimensions for the diagnosis of ND. The review included 88 studies. Most studies focused on Alzheimer’s disease and mild cognitive impairment. The literature remained predominantly centred on isolated diagnostic domains, and important limitations were consistently identified, including methodological heterogeneity, lack of standardized thresholds, and reduced clinical applicability. Based on the identified conceptual and methodological limitations, a Multidimensional Model of Neurocognitive Disorders (MOND model) for ND diagnosis was proposed. The MOND model was developed as a multidimensional, multilevel, transdiagnostic model integrating biological, neurocognitive, neuropsychiatric, motor, functional, frailty, reserve-related, and socio-environmental dimensions. The model may contribute to research, symptom classification, severity characterization, prognosis, and personalized intervention planning across different ND trajectories. Future studies using the MOND model should focus on refining algorithms to estimate the risk of ND.

## 1. Introduction

Neurocognitive Disorders (ND) are commonly diagnosed considering current versions of the International Classification of Diseases and Related Health Problems (ICD) by the World Health Organization [1] and the Diagnostic and Statistical Manual of Mental Disorders (DSM) by the American Psychiatric Association [2]. Additionally, several guidelines have been considered to differentiate between different types of Major ND (for an in-depth example, refer to the National Institute for Health and Care Excellence, [3]). Nonetheless, ICD and DSM have been criticized due to their categorical foundation that overlooks variations in symptom presentation, poor prognosis validity [4,5], inadequate coding regarding the severity of diseases [6], diagnostic instability [7], arbitrary boundaries between psychopathology and normality [7], and cases of unnoticed presence of comorbidity between disorders [8]. For example, the ICD-11 only includes mild cognitive impairment and dementia as organic mental disorders instead of considering the full range from cognitive healthy ageing to severe dementia. Even when these classification systems are used, it is recommended to perform individualized assessments addressing multiple causal factors [5].

Given the limitations of categorical disease classifications such as the ones above, two alternative frameworks have been proposed: the Research Domain Criteria (RDoC) framework by the National Institute of Mental Health (NIMH; [6,9] and, more recently, the Hierarchical Taxonomy of Psychopathology (HiTOP) [7]. Both frameworks adopt a dimensional approach to mental health problems [10]. This means that instead of categorizing disorders into distinct, separate categories, RDoC and HiTOP view mental health conditions along a spectrum of severity and a constellation of symptoms, which are frequently shared between diagnostic taxonomies. A dimensional approach can be applied to ND by considering neurocognitive impairments as existing on a *continuum*, encompassing diverse cognitive and behaviour manifestations, rather than being confined to well-defined and unique diagnostic categories. In fact, neurocognitive functions are an umbrella for several interdependent processes influenced by a variety of individual variables, which can include different comorbidities. Even a similar organic involvement can lead to a wide range of clinical symptom presentations at the neurocognitive and behavioural levels. Rigid diagnostic systems may hinder the ability to update case formulation and how ND progresses. This flexibility becomes especially critical when specific genetic or medical tests are unavailable to confirm brain injury or disease. In such cases, clinicians rely mostly on non-specific medical exams and neuropsychological assessment results for the clinical case formulation. A dimensional approach may allow for more adaptive and personalized assessments that can dynamically evolve as the clinical condition changes, offering a more nuanced understanding of ND over time.

### 1.1. Research Domain Criteria (RDoC)

The RDoC framework consists of a transdiagnostic approach with a special focus on psychopathology research [6,10]. This approach considers dimensional constructs at different levels of analysis, integrating elements from biology to psychology, encompassing genetics and neuroscience [6,10]. The RDoC also presents the potential to inform future classifications [10]. For example, in the domain of cognitive systems, the RDoC encourages the investigation of specific processes such as attention, working memory, and perception, all of which can be quantified along a continuum of functionality. This approach allows researchers and clinicians to explore the variability in neurocognitive functioning both within and between individuals, regardless of whether they meet the criteria for a specific disorder.

### 1.2. Hierarchical Taxonomy of Psychopathology (HiTOP)

The HiTOP model is also a research-based quantitative nosology, using factor analysis to classify psychopathological syndromes and their subtypes, considering the observed covariation of symptoms [7]. The HiTOP approach involves combining related symptoms as well as co-occurring syndromes [7]. The rationale behind the HiTOP is based on the premise that psychiatric conditions represent extensions of normal mental functioning. This approach works well for many psychiatric disorders that exist along a spectrum of normal to abnormal functioning. However, ND typically arises from distinct genetic, morphological, or pathological changes in the brain, rather than being extensions of normal cognitive processes. As a result, the HiTOP model may not fully capture the nature of ND, which often involves discrete biological events, rather than a functional continuum. That said, the HiTOP perspective still offers valuable inspiration for developing new models of ND. These conditions often follow a trajectory from a premorbid neurocognitive level through varying stages of impairment. Since premorbid ability can influence both the onset and progression of ND, a dimensional approach like HiTOP, namely one that tracks severity over time, can be adapted to capture these changes. This perspective highlights the potential to incorporate the influence of baseline neurocognitive functioning on disease progression, supporting a more nuanced understanding of ND.

### 1.3. Combining RDoC and HiTOP Frameworks to ND

Recently, a new proposal has emerged advocating for a synergistic combination of RDoC and HiTOP frameworks to develop a unified, biobehavioural nosology for the realm of psychiatry [10]. However, according to Ross and Margolis [11], both frameworks have limitations concerning ND. Specifically, Ross and Margolis argue that RDoC disregards the fact that ND have a distinct natural history and employs a top-down approach to research on mental illness, further dividing brain function into subsystems and sub-subsystems [11]. For ND, beginning with basic etiologic elements and analyzing how they may interact to lead to different syndromes may provide a better framework for understanding the emergence of different ND. Regarding HiTOP, it still needs to extend its classification system to include Major ND [12].

Although both RDoC and HiTOP have limitations in the context of ND, the shift from diagnosis-centred approaches to symptom-centred approaches seems to be promising in Major ND [13]. Indeed, the potential of transdiagnostic approaches to identify systematic, non-random variations in cognitive-behavioural symptoms among individuals with ND, regardless of their specific categorical diagnosis, is increasingly recognized [13,14]. This recognition has prompted the application of a transdiagnostic perspective to Major ND, namely to Major Frontotemporal ND [13,14].

Currently, there is no comprehensive framework that integrates the dimensional benefits of models like RDoC and HiTOP with the distinct biological, functional, and social realities of the broader ND. In daily practice, whereas RDoC fragments human function into sub-systems and HiTOP focuses on psychiatric covariation, a model applying a unified, dynamic profile that directly guides ND diagnosis to support personalized interventions is necessary (see Table 1). Therefore, the main objective of this work is to critically review the literature on ND diagnosis.

## 2. Method

This critical review aimed to synthesize and critically discuss the most significant literature on the diagnosis of ND, with particular emphasis on conceptual contributions, methodological approaches, and current limitations in the field. Although critical reviews do not necessarily require a fully systematic methodology, a structured search strategy was adopted to improve transparency and reduce potential selection bias.

The literature search was conducted in EBSCO (PsycInfo, PsycArticles, and Psychology and Behavioural Sciences Collection), Scopus, and PubMed. The following search strategy was used: “dementia OR neurocognitive disorder OR mild cognitive impairment” AND “diagnos*” in the titles. Searches were restricted to reviews and meta-analyses involving human participants published from 2013 onwards. This temporal criterion was defined considering the publication of the DSM-5 in 2013, which marked a significant shift in the conceptualization and diagnosis of cognitive impairment. After duplicates were removed, titles and abstracts were screened according to predefined eligibility criteria. Studies were included if they (a) were systematic reviews or meta-analyses and (b) focused on the diagnosis of ND.

## 3. Results

### 3.1. Critical Review

The critical review included 88 reviews, comprising systematic reviews (*n* = 50), systematic reviews with meta-analysis (*n* = 25), meta-analyses (*n* = 12), and one systematic review of systematic reviews (*n* = 1). The most frequently studied populations were individuals with AD (*n* = 45) and those with mild cognitive impairment (*n* = 40) (see Table 2). Regarding context/setting, 34 studies did not clearly specify the setting. Among the remaining studies, clinical/healthcare settings were the most frequent (*n* = 36). The review also highlighted substantial variability across levels of care, with primary care settings relying predominantly on brief cognitive screening and informant-based measures.

The critical review further highlighted that the current literature on ND diagnosis remains highly fragmented across partly isolated dimensions of analysis. Most reviews focused on individual diagnostic domains, particularly neuroimaging (*n* = 32), fluid biomarkers (*n* = 19), brief cognitive screening tools (*n* = 22), or artificial intelligence/machine learning approaches (*n* = 12), frequently evaluating these dimensions independently rather than within an integrated multidimensional framework. Although several approaches demonstrated promising diagnostic utility, particularly fluorodeoxyglucose-positron emission tomography (FDG-PET), amyloid/tau PET, dopamine transporter-Single Photon Emission Computed Tomography (DAT-SPECT), volumetric MRI, and fluid biomarkers such as p-tau181 and p-tau217, the review also identified important limitations related to methodological heterogeneity, lack of standardized thresholds, limited external validation, and reduced clinical applicability. For example, multimodal machine learning approaches integrating biomarkers, neuroimaging, and neuropsychological measures demonstrated promising results for improving diagnostic prediction in AD and MCI [16,21]; yet concerns regarding methodological heterogeneity and limited generalizability remained. Similarly, several reviews evaluating biomarker and neuroimaging approaches concluded that evidence was insufficient to support routine implementation in clinical practice [15,84]. Notably, the reviewed studies rarely discussed ND diagnosis within the context of DSM-5 or ICD and did not consider RDoC or HiTOP for ND classification or transdiagnostic analysis. Additionally, considering the complexity of ND, a multilevel diagnostic pathway was proposed that begins with the identification of cognitive complaints and exclusion of reversible causes in non-specialist settings, followed by increasingly specialized assessments aimed at differential diagnosis, subtype characterization, and individualized management [37].

The main conclusion of this review is that a significant gap persists between categorical diagnostic systems and the growing body of evidence on biomarkers and other disease-related indicators. Addressing this gap is particularly important, given the considerable clinical heterogeneity observed within individual ND, the overlap in neuropathological processes and biological alterations across different disorders, and the occurrence of mixed or atypical presentations. Moreover, most studies have predominantly focused on individual biological or psychological domains, with limited efforts to integrate these dimensions into a comprehensive and clinically actionable diagnostic model.

### 3.2. The MOND Model: A Multidimensional Model of ND

The recognition that ND cannot be fully understood through purely categorical or exclusively biomedical frameworks is not entirely new. Previous proposals have already emphasized the importance of multidimensional and biopsychosocial perspectives in ND care and clinical formulation. For example, the Biopsychosocial (BPS) model of ND proposed by Spector and Orrell [104] emphasized the interaction between biological, psychological, and social processes across the trajectory of ND, distinguishing between fixed and tractable factors that may influence symptom expression and intervention response. Subsequently, preliminary evidence suggested that training healthcare professionals to apply the BPS model improved formulation skills and the ability to develop individualized interventions for people with ND. These findings reinforced the potential clinical utility of multidimensional and formulation-based approaches in ND care [104].

However, despite their important contributions, this model remained primarily focused on ND care and psychosocial formulation, rather than providing a comprehensive multidimensional and transdiagnostic model for ND diagnosis. A Multidimensional Model of ND (MOND model) was therefore developed to extend these perspectives into a unified multidimensional framework intended to support ND diagnosis.

Multidimensionality in this context extends the traditional clinical approach of isolated symptom tracking or simple comorbidity counting. Here, it is defined as the continuous, interactive, and non-linear influence of biological, clinical-functional, and socio-environmental variables across the entire disease spectrum. Rather than viewing these domains as parallel or independent tracks, a multidimensional perspective captures how they dynamically modulate one another to dictate each individual clinical trajectory.

**Figure 1:**
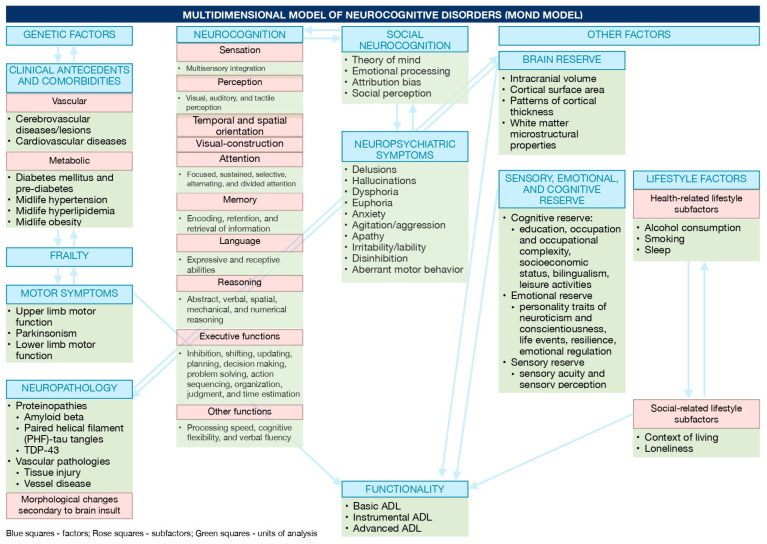
Multidimensional model of Neurocognitive Disorders (MOND model).

The MOND model proposed here is organized into factors, subfactors, and units of analysis. The selection of these specific factors was not arbitrary; it was driven by a strict inclusion criterion. To be included as a primary factor in the MOND model, a variable must possess established empirical evidence demonstrating its capacity to modulate the onset, phenotypic presentation, or progression trajectory of neurocognitive decline. Rather than subsuming all clinical manifestations under broad functional umbrellas, variables with distinct pathophysiological or temporal timelines were deliberately separated to maintain diagnostic and prognostic sensitivity. A primary example of this is the classification of motor symptoms. While traditionally grouped under general neurofunctional domains, motor symptoms are treated here as an independent factor. This structural decision reflects the clinical reality that in several major ND, such as Parkinson’s disease or Lewy Body Dementia, motor dysfunction frequently precedes or parallels cognitive decline. In these presentations, motor alterations act as distinct trajectory markers that require independent assessment and tailored clinical management, justifying their standalone position within the multidimensional model. The model encompasses the following factors: (a) genetic factors; (b) clinical antecedents and comorbidities; (c) frailty; (d) motor symptoms; (e) neuropathology; (f) neurocognition; (g) social neurocognition; (h) neuropsychiatric symptoms; (i) functionality; (j) other factors (see Figure 1). Figure 1 supports clinical case formulation and can act as a foundation for structuring a clinical interview framed on the MOND model.

Concerning *genetic factors*, both risk (e.g., two or more family members with the disease) and protective factors for ND (e.g., apolipoprotein ε2 and ε3 allele) should be considered [105,106]. Additionally, it is essential to provide information on whether the genetic factors are primary or secondary to brain insult or disease to ensure the adequacy of this model for conditions with a clear neurological basis. In relation to *clinical antecedents and comorbidities*, it is important to consider both *vascular*, *metabolic*, and *other* subfactors [106]. The units of analysis for the *vascular* subfactor should include cerebrovascular diseases/lesions, such as atherosclerosis and cerebral macrovascular and microvascular lesions [107,108,109,110,111], as well as cardiovascular diseases [112,113,114]. Similarly, the units of analysis for the *metabolic* subfactor should comprise variables such as diabetes mellitus and pre-diabetes, midlife hypertension, midlife hyperlipidemia, and midlife obesity [106,115], as well as hormonal causes (e.g., hypothyroidism). Inflammatory brain diseases, traumatic damage, and toxic poisoning of the Central Nervous System or tumours are other units of analysis. *Frailty* has also been recognized as a contributing risk factor for ND [116]. Frailty can be defined as a clinical syndrome characterized by the presence of three or more of the following criteria: unintentional weight loss; exhaustion; low physical activity; slow walking speed; and low grip strength [117]. The interplay between frailty and cerebrovascular diseases/lesions has been noted [118,119], as well as its association with cardiovascular diseases, increasing the risk of adverse outcomes in cardiovascular patients and, in turn, accelerating *frailty* [120]. Frailty also has a connection with the motor symptoms of ND [121]. In terms of the *motor symptoms* factor, three main motor domains were linked to the risk of Major ND and could be considered as units of analysis: upper limb motor function; lower limb motor function; and Parkinsonism [122]. Half-side problems and whole-body problems should also be considered, as well as tremor, ataxia, akinesia, ballism, and choreaform movements. Concerning lower limb motor function, gait has garnered particular attention due to its relationship with neurocognition and the risk of falls [123]. Additionally, motor symptoms are associated with the level of neurocognitive decline [124] and contribute to the differentiation between various types of ND [125]. Recent evidence also suggests that motor markers may contribute to ND identification and staging. For example, hand dexterity measures differentiated healthy older adults from AD patients and were associated with cognitive performance, while complex motor analyses may help predict progression from MCI to AD [69].

Concerning *neuropathology* factors, ND may involve proteinopathies such as amyloid beta, paired helical filament (PHF)-tau tangles, TDP-43, and/or vascular pathologies mostly caused by ischemic tissue injury due to brain infarcts, hypoxia, or hemorrhage with or without vessel disease, and hippocampal sclerosis [126]. This is consistent with findings from the critical review, which identified biomarkers associated with amyloid, tau, neurogranin, alpha-synuclein, neurofilament light chain, inflammatory pathways, and exosome-derived markers across different ND [26,55,81,86,100]. Importantly, several reviews reported that biomarker findings were not fully specific to a single diagnostic category, highlighting the complexity of linking individual biomarkers to specific ND. For example, CSF alpha-synuclein differentiated Lewy Body ND from Alzheimer’s Disease but not from Parkinson’s Disease ND or other neurodegenerative conditions [55]. *Morphological changes secondary to brain insult* may be considered a subfactor of neuropathology. Additionally, the protective effect of some hormones (e.g., estrogen) should also be considered. The role of *brain reserve* factor, defined as the anatomical or structural characteristics of the brain that enable individuals to cope with brain ageing or neuropathology better than others prior to the onset of neurocognitive changes [127], should be considered in the *other factors*. Contemporary perspectives emphasize that reserve emerges from the dynamic interaction between structural integrity, neural connectivity, and neurophysiological activity, rather than from isolated anatomical properties alone [128,129]. In this sense, preserved structural integrity and white matter organization have been associated with maintenance of functional synchrony, neural connectivity, and cognitive performance across ageing and ND [128]. Accordingly, the MOND model conceptualizes brain reserve as a multidimensional and dynamically interacting factor linked to cognitive, emotional, sensory, and functional domains.

The factor *neurocognition* can be better described considering the assessment of each of the neurocognitive functions: (a) sensation, including multisensory integration tasks; (b) visual perception (e.g., colour perception, face recognition, visual discrimination), auditory perception (e.g., speech perception, nonverbal auditory perception, auditory discrimination), and tactile perception (e.g., graphesthesia and stereognosis); (c) temporal and spatial orientation; (d) visual-construction; (e) attention, including focused, sustained, selective, alternating, and divided attention; (f) memory, including encoding, retention, and retrieval of visual, auditory or multisensory information, retrieval of episodic, semantic or procedural memory, and prospective memory; (g) language, including expressive abilities (i.e., semantic, written language, syntax, naming, pragmatic, repetition of words or sentences, discourse, lexical knowledge) and receptive abilities (i.e., reading and auditory comprehension); (h) reasoning, including abstract, verbal, spatial, mechanical, and numerical reasoning; (i) executive functions, including inhibition, shifting, updating, planning, decision-making, problem solving, action sequencing, organization, judgement, and time estimation; (j) other functions, including processing speed, cognitive flexibility, and verbal fluency [130].

The critical review further supports this multidimensional assessment approach. Several reviews demonstrated diagnostic utility for memory measures [22,98], spatial orientation tasks [27], and executive and visuospatial measures [53]. These findings suggest that no single cognitive domain fully captures ND-related impairment. Therefore, all neurocognitive functions should be assessed, bearing in mind that although some deficits are more common and characteristic of certain types of ND (e.g., episodic memory deficits in Alzheimer’s disease and personality changes in the behavioural variant of frontotemporal ND), different ND may also be expressed in similar performance in several neurocognitive functions (e.g., processing speed in Alzheimer’s disease and vascular dementia) [131]. It is therefore important to consider deficits in other domains that may impact functionality beyond the most common deficits observed in each type of ND. This includes, for example, deficits in functional communication, fluency, and sentence comprehension in the genetic forms of frontotemporal ND [132]. Moreover, the *social neurocognition* factor warrants consideration as a multidimensional domain of neurocognition [133]. Supporting this perspective, Dodich [34] reported that emotion recognition and theory of mind tasks demonstrated promising diagnostic utility in the behavioural variant of the frontotemporal ND, although evidence remains insufficient to recommend specific tasks for routine clinical use. Components of social neurocognition (i.e., theory of mind, emotional processing, attribution bias, and social perception) are affected differently by distinct ND [134]. Deficits in social neurocognition contribute to impairments in everyday social neurocognition functioning among individuals with ND [135], as well as socially inappropriate behaviours and neuropsychiatric symptoms [136]. Among the *neuropsychiatric symptoms* observed in ND, stands out: delusions; hallucinations; dysphoria; mania or an exaggerated and inappropriate euphoria; anxiety; agitation/aggression; apathy; irritability/lability; disinhibition; anosognosia; and aberrant motor behaviour. Specifically, affective neuropsychiatric symptoms such as depression, anxiety, apathy, and irritability can mask ND, a phenomenon referred to as pseudodementia [137,138]. These symptoms can also contribute to an increased risk of Major ND [139], as well as intensify as Major ND progresses [140]. Additionally, neuropsychiatric profiles appear to vary among different types of ND and levels of severity [141,142]. The importance of neuropsychiatric manifestations for diagnosis was also highlighted in the critical review. For example, Jreige [52] suggested that neuropsychiatric symptoms should be considered alongside dopaminergic imaging markers in the identification of prodromal Lewy Body ND, while Miller [68] provided consensus diagnostic criteria for apathy in ND.

The *functionality in the activities of daily living* consists of the hallmark of Major ND [143]. The units of analysis of this factor comprise: basic activities of daily living (e.g., urinating independently); instrumental activities of daily living (e.g., managing finances); and advanced activities of daily living (e.g., anticipating unforeseen events and finding alternatives) [144]. The impairment in these activities appears to represent a continuous spectrum as the severity of ND progresses [143,145,146].

The section on *other factors* was included to address influences that may affect certain conditions, such as ageing and Major ND, but may have a minimal impact in cases of significant brain injury or genetic etiology. The *other factors* include Brain reserve, *Lifestyle factors*, as well as *Sensory, Emotional, and Cognitive Reserve.* These factors are conceptualized as modulatory mechanisms within the MOND model, as they may influence the expression, severity, and progression of neurocognitive, emotional, social neurocognitive, and affective/neuropsychiatric manifestations. This interpretation is consistent with multidimensional approaches suggesting that reserve-related and frailty-related factors can shape the relationship between underlying neuropathology, clinical presentation, and prognosis in ND [147]. In line with these perspectives, recent models further emphasize that ageing-related vulnerability emerges from the dynamic interaction between intrinsic factors (e.g., genetic susceptibility) and extrinsic factors (e.g., lifestyle), which together may influence resilience, homeostatic balance, and disease trajectories [148]. Accordingly, reserve-related constructs in the MOND model are not conceptualized as isolated variables, but rather as cross-cutting mechanisms that may modulate multiple domains simultaneously.

Regarding the *lifestyle*, two main subfactors are proposed: health-related lifestyle factors, which comprise alcohol consumption, smoking [106], and sleep as units of analysis [149,150,151]; and social-related lifestyle factors, which include the context of living and loneliness as units of analysis. Concerning the context of living, previous studies showed that older adults living in nursing homes demonstrate a higher likelihood of experiencing neurocognitive decline compared to those who age within the community [152,153]. Furthermore, it is important to consider the conditions that facilitate ageing-in-place [154] and cognitive ability, which means the extent to which a community supports the cognitive health of ageing residents [155]. Loneliness constitutes a modifiable factor and has also been demonstrated to increase the susceptibility to ND [156]. Specifically, persistent loneliness experienced during midlife was linked to atrophy in brain regions implicated in memory and executive function [157]. In this sense, lifestyle and social-related factors should not be interpreted as isolated variables, but rather as dimensions that interact with biological, psychological, cognitive, and functional processes within a multidimensional model of frailty and ND [158]. These interactions may contribute to differences in functional autonomy, cognitive decline, and vulnerability to adverse neurocognitive outcomes over time [158].

The *Sensory, Emotional, and Cognitive Reserve* may be based on a model in which cognitive reserve (i.e., the adaptability of cognitive processes that contribute to different individual susceptibility of neurocognition to brain ageing or neuropathology compared to others) [127], sensory reserve (i.e., “the adaptability of sensory processes that help to explain the individual differences in the processing of sensory information when facing sensory-related disease or sensory deprivation”, p. 4), and emotional reserve (i.e., “adaptability of the individual emotional functioning, which helps to explain individual differences in affective functioning, as well as in the processing of emotional information when facing emotionally challenging situations”, p. 5) interact with each other, thereby postponing the manifestation of neurocognitive deficits in terms of functionality in the instrumental cognitive activities of daily life [159]. ND appear to exhibit distinct neurocognitive profiles [160,161,162], and the factor *neurocognition* may be influenced by cognitive reserve [159].

### 3.3. Guidelines for Translational Research with the MOND Model

The translation of the MOND model to practice requires some steps to be followed: (1) identifying scales/instruments for assessing each factor; (2) creating a collaborative multidisciplinary semi-structured interview; (3) preliminary testing of participants following the model; (4) mediation and moderation analyses of different factors; (5) refining the model based on the results of moderation analysis; (6) defining decision trees to follow in the diagnosis and prognosis using MOND model. The guidelines for translational research and clinical application of the Model are provided in Table 3.

Mediation and moderation analyses will play a critical role in the context of the MOND model, specifically to identify and understand how various factors interact with each other and influence the severity and progression of ND. In the MOND model, multiple factors are included. All may play a role in the development and severity of ND, but it is important to identify which ones may significantly cause, influence, or characterize the different ND and understand how these influences occur. These analyses allow for the exploration of how these factors may act independently and also how their impact on ND outcomes may change depending on the presence or level of other factors. For example, a moderation analysis could help clarify whether the relationship between neurocognition and functional decline is stronger in individuals with certain comorbidities (e.g., vascular issues or metabolic disorders). Identifying interaction effects of this type will contribute to a more nuanced understanding of the ND and aid in personalizing treatment and intervention strategies based on individual profiles.

The translational value of the MOND model is particularly evident when its multidimensional framework is operationalized through advanced computational methods, such as machine learning. For instance, recent predictive modelling in cardiovascular populations demonstrates that neurocognitive outcomes following an Acute Coronary Syndrome cannot be accurately forecasted by isolated variables. Instead, highly accurate prognostic models require the integration of specific MOND factors. Strikingly, in these models, neuropsychiatric symptoms, specifically depression, emerged as the second most powerful predictor of cognitive outcomes, operating alongside clinical comorbidities (e.g., BMI, waist circumference) and socio-demographic reserve markers (e.g., age, schooling) [163]. This type of empirical evidence strongly validates the MOND framework’s core premise: by systematically mapping the non-linear interactions between biological, clinical, and neuropsychiatric factors, researchers can develop robust predictive algorithms.

Additional future studies on MOND model should focus on refining algorithms to achieve a more precise estimation of ND risk [164], developing personalized prognoses and treatments as proposed for other conditions marked by varying clinical trajectories among patients [165,166], developing a methodology to assess the severity of Major ND according to MOND model, and refining the model in order to improve their usefulness in clinical practice. Studies are being conducted by a team of neuropsychologists and neurologists to propose a clinical interview framed in the MOND model and to identify specific measures within the relevant factors that can be assessed behaviourally. Although cognitive measures are relevant for elucidating complex cases of ND and performing fine-grained assessments between and within levels of severity, the evidence indicates that such measures are of limited value for differential diagnosis. Consequently, our research is also focused on identifying qualitative and quantitative patterns of impaired cognitive processes across different forms of ND, as well as patterns of normal functioning in older adults [131].

## 4. Discussion

The main objective of this work was to critically examine the evidence on ND diagnosis. Overall, the findings revealed a predominantly biomarker- and cognition-oriented literature characterized by fragmentation across diagnostic domains, methodological heterogeneity, limited clinical translation, and insufficient integration of biological, psychological, and social dimensions within a unified diagnostic model. Thus, the resulting knowledge was integrated into a multidimensional and multilevel model (MOND model) to support Neurocognitive Disorders (ND) diagnosis. The MOND model integrates biological, neurocognitive, neuropsychiatric, motor, functional, frailty, reserve-related, and socio-environmental dimensions.

The main contribution of this manuscript lies in developing a more comprehensive multidimensional model that tentatively fills the gaps left by RDoC and HiTOP in the context of ND. The MOND model may also offer a structured framework to help in research, symptom classification, assessment of ND severity and its prognosis, as well as intervention. A key strength of this model is its more holistic approach towards the complex nature of ND, considering its heterogeneity and dimensional aspects tied to the varying severity of symptoms. As such, the MOND model may enable a more complete identification of symptoms across the spectrum from subclinical ND to severe Major ND. Additionally, this model follows a transdiagnostic approach, including the core symptoms to differentiate among various types of ND [126], also acknowledging that different types of ND are often mixed [126]. Therefore, it must be highlighted that there is no single behavioural marker capable of consistently discriminating different ND [167]. Of note, this proposal is in its initial stages and does not exhaustively describe each factor, subfactor, and unit of analysis.

The main limitations of this work are the absence of a formal quality assessment of the studies included in this critical review and the lack of empirical validation of the MOND model. Consequently, the findings should be interpreted considering the methodological heterogeneity of the included reviews. Nevertheless, guidelines are proposed to facilitate the future operationalization, empirical validation, and clinical implementation of the MOND model.

Future studies using this model are expected to work on identifying scales/instruments for assessing each factor, refining algorithms to achieve a more precise estimation of ND risk [164], developing personalized prognoses and treatments as proposed for other conditions marked by varying clinical trajectories among patients [165,166], developing a methodology to assess the severity of Major ND according to MOND model, and refining the model in order to improve their usefulness in clinical practice.

Studies are being conducted by a team of neuropsychologists and neurologists to propose a clinical interview framed in the MOND model and to identify specific measures within the relevant factors that can be assessed behaviourally. Although cognitive measures are relevant for elucidating complex cases of ND and performing fine-grained assessments between and within levels of severity, the evidence indicates that such measures are of limited value for differential diagnosis. Consequently, our research is also focused on identifying qualitative and quantitative patterns of impaired cognitive processes across different forms of ND, as well as patterns of normal functioning in older adults [131]. Future validation studies, meta-analyses, and umbrella reviews examining the diagnostic accuracy and prognostic utility of neurocognitive assessment tools, biomarkers, and multidimensional indicators are essential to refine staged clinical workflows based on the MOND model, particularly regarding how assessments may be organized across sequential levels of care within multidisciplinary healthcare settings.

## 5. Conclusions

The reviewed literature revealed important gaps between research and clinical practice, including the fragmentation of evidence across individual diagnostic domains, limited integration of biological and psychological indicators, lack of multidimensional frameworks, and insufficient translation of promising diagnostic advances into routine clinical decision-making. The MOND model is proposed as a comprehensive diagnostic framework that can be applied in a stepwise manner across different healthcare settings to support early ND diagnosis. By emphasizing the characterization of the individual’s multidimensional clinical profile rather than requiring immediate attribution to a specific ND subtype, the model may help reduce diagnostic uncertainty. Although differential diagnosis remains clinically relevant, symptom presentation and the overall clinical profile continue to play a central role in clinical decision-making.

## Figures and Tables

**Table 1:** Pros and cons of disease classification systems in the realm of neurocognitive disorders.

System	Pros	Cons
Diagnostic and Statistical Manual of Mental Disorders (DSM)	- Facilitates clinical diagnosis and communication among healthcare professionals due to its widespread adoption.	- The categorical system may oversimplify the complexity of neurocognitive disorders. - Criteria may not fully capture subthreshold or early cognitive impairments. - Lacks emphasis on neurobiological underpinnings and dimensions of functioning. - Of limited usefulness from a research perspective.
International Classification of Diseases (ICD)	- Facilitates clinical diagnosis and communication among healthcare professionals due to its widespread adoption.	- Oversimplify conditions and reduce the emphasis on underlying mechanisms.
Research Domain Criteria (RDoC)	- Focuses on underlying biological, genetic, and neurological factors, offering a more in-depth understanding of neurocognitive disorders. - Encourages research into neurobiological mechanisms, which can lead to more targeted treatments. - Transdiagnostic approach, avoiding strict categories, facilitating flexible exploration of neurocognitive symptoms.	- Lacks specific diagnostic criteria, making it difficult to use in clinical practice for diagnosis. - Primarily research-focused, less applicable for direct clinical management.
Hierarchical Taxonomy of Psychopathology (HiTOP)	- Captures comorbidities and symptom overlaps often seen in neurocognitive disorders. - More personalized, patient-centred approach.	- Still under development, with less clinical utility at present. - Limited data and research on applying HiTOP specifically to neurocognitive disorders.

**Table 2:** Characteristics of studies included in the critical review.

No.	First Author	Study Type	Population	Context/Setting	Diagnosis Criteria—Instruments/Measures	Main Results
1	Arevalo-Rodriguez [15]	Systematic review	AD	Clinical setting	Diagnostic recommendations on AD dementia Management of patients with dementia: A national clinical guideline Management of dementia Clinical practice guidelines about comprehensive care for people with Alzheimer’s disease and other dementias EFNS guidelines for the diagnosis and management of Alzheimer’s disease	Diagnostic AD dementia guidelines—methodological heterogeneity. Brief cognitive tests, particularly MMSE, were commonly recommended for initial assessment. Neuropsychological evaluation was recommended mainly when the diagnosis was unclear or when a differential diagnosis was needed. Most guidelines did not support routine use of biomarkers in clinical practice due to limited evidence and standardization concerns. Standardized, evidence-based diagnostic frameworks for AD dementia are needed.
2	Blanco [16]	Systematic review	MCI, AD	NA	CSF, blood, and saliva biomarkers combined with machine learning algorithms, neuroimaging, and neuropsychological measures.	Multimodal machine learning models integrating biomarkers and neuroimaging showed promise for improving diagnostic prediction of AD in MCI populations.
3	Borchert [17]	Systematic review	MCI, AD, PD, VaD, LBD, FTD, PSP, HD, CBD	Clinical setting, training dataset	Machine learning models applied to MRI, PET, SPECT, EEG, MEG, and ultrasound	Improved diagnostic accuracy when a combination of neuroimaging modalities was used. Findings suggest superior performance of discriminative models compared to algorithmic and generative classifiers for the classification of AD vs. healthy controls. Limitations of artificial intelligence—lack of sufficient algorithm development descriptions and standard definitions.
4	Breton [18]	Meta-analysis	MCI	Community and secondary care settings	Cognitive tests	ACE-R, CERAD, MoCA, and Qmci—similar diagnostic accuracy MMSE—lower sensitivity. Memory Alteration Test—highest sensitivity and equivalent specificity to the other tests.
5	Brigo [19]	Systematic review and meta-analysis	AD, FTD, LBD	NA	123I-FP-CIT SPECT (DaTSCAN), visual and semiquantitative analyses	123I-FP-CIT SPECT showed sensitivity and specificity values above 80% for differentiating DLB from other dementia syndromes, supporting its diagnostic utility.
6	Carnero-Pardo [20]	Systematic review and meta-analysis	Dementia	Different settings	Phototest	Phototest: - Sensitivity—0.85 - Specificity—0.87
7	Catino [21]	Systematic review	MCI, AD	Healthcare settings	Multimodal biomarker AI techniques—Diagnostic models (MCI/AD vs. normal), Prognostic models (MCI → AD conversion) MRI, PET, CSF, Plasma, Cognition, APOE, Retina imaging	Multimodal AI models (integrating two or more data types—neuroimaging, fluid biomarkers, retinal imaging, cognitive measures) generally outperformed unimodal models for early diagnosis and risk stratification of ND. MRI + CSF and MRI + PET combinations demonstrated particularly robust performance for AD/MCI classification. Limited external validation and methodological heterogeneity reduced generalizability and clinical translation.
8	Chan [22]	Systematic review and meta-analysis	MCI, AD	Any kind of setting	Verbal and visual memory tests, paper-and-pencil tests	Both verbal and visual computerized memory tests have comparable diagnostic performance to the paper-and-pencil tests.
9	Chan [23]	Systematic review	MCI, dementia	NA	Digital cognitive tests	Most digital cognitive tests demonstrated diagnostic performance comparable to traditional paper-and-pencil tests. Several tests showed good sensitivity and specificity for detecting MCI or dementia, supporting their utility for accessible and repeated cognitive screening. Digital tests—only had a few validation studies to verify their performance.
10	Chan [24]	Systematic review and meta-analysis	MCI, dementia	Clinical or community settings	Digital Drawing Tests and Paper-and-Pencil Drawing Tests	The digital clock drawing test—better diagnostic performance than paper-and-pencil format for MCI. Other types of digital drawing tests showed comparable performance with paper-and-pencil formats. Digital drawing tests can be used as an alternative tool for the screening of MCI and dementia.
11	Chen [25]	Systematic review and meta-analysis	MCI, dementia	Primary care setting	AD8	AD8 score ≧ 2—highly suspected to have cognitive impairment, and a further definite diagnosis is needed.
12	Chen [26]	Meta-analysis	Amnestic MCI, AD	Research settings	Blood biomarkers	For differentiating patients with AD from the controls: - Plasma Aβ42 (sensitivity = 88%, specificity = 81%) - Plasma Aβ oligomer (sensitivity = 80%, specificity = 88%) - Plasma tau (sensitivity = 90%, specificity = 87%). For differentiating aMCI from the controls: - Plasma Aβ42 (sensitivity = 86%, specificity = 90%) - Plasma tau (sensitivity = 79%, specificity = 94%). Blood-based biomarkers are minimally invasive and cost-effective tools for detecting AD.
13	Costa [27]	Systematic review	MCI	Clinical setting	Spatial orientation tasks, including questionnaires, paper-and-pencil tests, route learning, virtual reality, and computer-based tasks	Spatial orientation tasks—moderate-to-high diagnostic accuracy for distinguishing MCI from healthy ageing, with AUC values ranging from 0.77 to 0.99.
14	Custodero [28]	Systematic review and meta-analysis	VD	NA	Inflammatory markers—interleukin (IL)-6, C-reactive protein (CRP), tumour necrosis factor (TNF)-α] from blood or CSF	Blood IL-6 levels > people with VD compared to AD patients and controls—might represent a useful biomarker able to differentiate people with VD from those with AD
15	Custodio [29]	Systematic review	MCI or dementia	Latin America Spanish speaking population Different settings (memory clinic-based samples, adult day-care centre, primary care clinic, and community-based sample	Brief cognitive screening tools	Most studies used adequate diagnostic accuracy measures. Brief cognitive screening tools: - Differed in terms of the cognitive domains evaluated. - Validated in a memory clinic setting - Limited description of the psychometric properties of the instruments
16	Dagher [30]	Systematic review and meta-analysis	FTD	NA	ASL-MRI and FDG-PET	ASL MRI—sensitivity of 0.70 and specificity of 0.81 [18F]-FDG-PET—sensitivity of 0.88 and specificity of 0.89
17	Dattola [31]	Systematic review	Frontotemporal dementia	NA	Artificial intelligence approaches in differential diagnosis	ML techniques—potential for improving FTD diagnosis. Support Vector Machines (SVMs)—applied to neuroimaging and electrophysiological data. Deep learning methods—high accuracy in distinguishing FTD from other dementias. Multimodal data, including neuroimaging, EEG signals, and neuropsychological assessments, enhance diagnostic accuracy.
18	Davison [32]	Systematic review	MCI, AD, LBD, FTD	NA	PET, SPECT	PET studies generally report higher accuracy for AD than SPECT—evidence based on direct PET and SPECT comparison studies is limited
19	del Campo [33]	Systematic review	FTD	NA	CSF and plasma biomarkers, NfL, TDP-43, tau biomarkers, genetic-related fluid biomarkers	The clinicopathological heterogeneity of FTD limits accurate diagnosis based only on clinical phenotype. Biofluid biomarkers—may improve diagnosis, disease staging, and prediction of underlying pathology. NfL—one of the most promising biomarkers of FTD
20	Dodich [34]	Systematic review	Behavioural variant of FTD	Clinical settings	Social cognition measures	Major risk of bias—lack of pathological confirmation. Evaluation of the accuracy of social cognition tasks in bvFTD—mainly focused on emotion recognition and ToM. Emotion recognition and ToM tasks—could be the best choice to ensure a high diagnostic accuracy in clinical settings. No recommendation concerning the use of a specific social task in bvFTD diagnosis can currently be provided
21	Donaghy [35]	Systematic review and meta-analysis	MCI with Lewy bodies	NA	Diagnostic criteria for mild cognitive impairment with Lewy bodies (McKeith et al. [36])	The meta-analysis supported the inclusion of the current clinical features in the diagnostic criteria. Quantitative EEG and fluorodeoxyglucose PET—promise as diagnostic biomarkers.
22	Fabrizi [37]	Systematic review	MCI, AD, LBD, FTD, VD	Non-specialist and specialist clinical settings—Italy’s first National Guideline on dementia and MCI diagnosis	Non-specialist setting: clinical history (including informant history), physical examination, blood/urine tests, cognitive screening (10-CS, 6CIT, 6-IS, MIS, Mini-Cog, TYM, GPCOG), CT/MRI to exclude reversible or secondary causes. Specialist setting: neurological examination, neuropsychological assessment, validated subtype criteria [International consensus criteria for dementia with LBD; International FTD criteria for frontotemporal dementia (primary non-fluent aphasia and semantic dementia); International FTD Consortium criteria for behavioural variant FTD; NINDS-AIREN criteria for vascular dementia; NIA-AA criteria for Alzheimer’s disease; Movement Disorders Society criteria for Parkinson’s disease dementia; WHO and International criteria for Creutzfeldt–Jakob disease], further tests for Alzheimer’s disease (PET/perfusion SPECT, CSF biomarkers for suspected AD), further tests for dementia with LBD (I-FP-CIT SPECT, I-MIBG cardiac scintigraphy, polysomnography with EEG), further tests for frontotemporal dementia (F-FDG PET or perfusion SPECT), further tests for vascular dementia (MRI, if MRI is unavailable or contraindicated, use CT), distinguishing dementia from dementia with delirium or delirium alone (confusion assessment method (CAM); • 4-A’s Test (4AT).	The guideline recommends a stepwise diagnostic pathway, beginning with detection and exclusion of reversible causes in non-specialist settings, followed by specialist assessment for subtype diagnosis and tailored management.
23	Fernandes [38]	Systematic review of systematic reviews	Dementia	Primary care	Cognitive tests	Mini-Cog and MMSE—most widely studied cognitive screening tools. AMTS—high sensitivity 100%, specificity 82%, within the shortest amount of time, within primary care.
24	Forlenza [39]	Systematic review	MCI, AD	Research and clinical settings	CSF biomarkers, structural and functional neuroimaging, multimodal biomarkers.	Clinical-biological insights led to the identification of the AD signature in the CSF. Methodological limitations still restrict its widespread clinical application.
25	Fornari [40]	Systematic review	Dementia	Clinical and research settings	Visual rating scales of brain atrophy	All scales—fair to excellent level of inter- and intra-rater agreement. Negative correlations between the rating in each scale and brain volumetric measures. Discriminative abilities—variability according to the scale and the population comparisons. Visual rating scales of atrophy—a reliable method for distinguishing physiological ageing from pathological conditions, and among neurodegenerative forms.
26	Haidar [41]	Systematic review and meta-analysis	Dementia	NA	ASL-MRI versus FDG-PET	FDG-PET—better than ASL-MRI, with pooled sensitivity being significantly higher for FDG-PET
27	Harrison [42]	Systematic review	Dementia	General practice (primary care) setting	IQCODE	IQCODE Cut-off—3.2 sensitivity 100%, specificity 76%; Cut-off—3.7 sensitivity 75%, specificity 98%. It is not possible to give definitive guidance on the test accuracy of IQCODE for the diagnosis of dementia in a primary care setting based on the single study identified
28	Harrison [43]	Systematic review	Dementia	Secondary care setting	IQCODE	IQCODE: - Cut-off of 3.3—sensitivity 0.91 and specificity 0.66 - Performs better in a ‘general’ setting.
29	Harrison [44]	Systematic review	Dementia	A variety of healthcare settings	IQCODE	IQCODE: - Cut-off of 3.3—sensitivity 0.84 and specificity 0.87 for the clinical diagnosis of dementia at one year after stroke. No specific recommendations on the use of the IQCODE in clinical practice are provided.
30	Howe [45]	Systematic review and meta-analysis	MCI and AD	Research setting	Auditory P300 latency	P300 latency—significantly prolonged in patients with AD (and MCI) compared to unaffected controls. Shortened P300 latencies were observed when comparing patients with MCI to patients with AD. Auditory P300 latency—a biological marker of prodromal AD.
31	Hsu [46]	Meta-analysis	MCI/mild dementia	Clinical setting	VCAT	VCAT: - Acceptable diagnostic accuracy in distinguishing MCI/mild dementia in cognitively normal older adults. Language-neutral and culturally unbiased tool
32	Huo [47]	Systematic review and meta-analysis	Dementia	Clinical setting (Chinese population)	Dementia screening tools	MMSE—sensitivity of 0.87 and specificity of 0.89 ACE-R—sensitivity of 0.96 and specificity of 0.96 MoCA—sensitivity of 0.93 and sensitivity of 0.90 GPCOG, Hasegawa’s Dementia Scale, and Cognitive Abilities Screening Instrument—performances comparable to that of the MMSE
33	Hwang [48]	Systematic review	MCI, AD, VD, LBD, FTD	Hospital setting	Six-Item Cognitive Impairment Test, Cognitive Performance Scale, Clock-Drawing Test, MMSE, and Time and Change test	There is insufficient evidence to recommend for or against the use of a specific test for screening for dementia or MCI in older hospital inpatients. Simple cognitive tests used in isolation are not reliable enough
34	Ibrahim [49]	Systematic review	MCI, AD	NA	Resting-state fMRI for detection of network connectivity	Multimodal support vector machine (SVM) algorithm—commonest form of ML method utilized.
35	Jafari [50]	Systematic review and meta-analysis	MCI	NA	Speech-based biomarkers using ASR technologies, NLP models, ML algorithms	Speech analysis—pooled accuracy 80%, sensitivity 80%, specificity 77%, and AUC 78% for distinguishing MCI from cognitively unimpaired individuals
36	Jiang [51]	Meta-analysis	MCI, AD	NA	ERP P300	MCI—P300 latency differed from controls and AD. P300 latency—may be a sensitive indicator for early cognitive decline
37	Jreige [52]	Systematic review	DLB	NA	Functional dopaminergic scintigraphic imaging	Early diagnosis—could be facilitated by identifying the prodromes of LBD using dopaminergic scintigraphic imaging coupled with emphasis on clinical neuropsychiatric symptoms.
38	Julio-Ramos [53]	Systematic review	AD, LBD	Research setting	Neuropsychological assessment of memory, executive function, attention, visuospatial/visuoconstructive abilities, and verbal fluency, alongside neuroimaging correlates	Differential diagnosis between AD and DLB remains challenging. Specific neuropsychological markers combined with anatomical and functional correlates may improve diagnostic accuracy.
39	Lees [54]	Systematic review	Multidomain MCI and dementia in stroke	Clinical setting	MMSE; MoCA; ACE-R; Rotterdam-CAMCOG	Common cognitive screening tools—similar overall diagnostic accuracy for post-stroke dementia/multidomain cognitive impairment. MoCA—high sensitivity but lower specificity at standard cut-offs. ACE-RR (cut-off < 88/100): sensitivity 0.96, specificity 0.70. MMSE (cut-off < 27/30): sensitivity 0.71, specificity 0.85. MoCA (cut-off < 26/30): sensitivity 0.95, specificity 0.45; (cut-off < 22/30): sensitivity 0.84, specificity 0.78 Rotterdam-CAMCOG (cut-off < 33/49): sensitivity 0.57, specificity 0.92 No single screening test clearly outperformed the others.
40	Lim [55]	Systematic review and meta-analysis	AD, LBD, PDD	NA	CSF alpha-synuclein	Mean CSF alpha-synuclein concentration was significantly lower in DLB patients compared to those with AD. No significant difference was found between patients with DLB compared to PDD or other neurodegenerative conditions. CSF alpha synuclein—may be of diagnostic use in differentiating between DLB and AD.
41	Ling [56]	Systematic review and meta-analysis	AD, VD	NA	Inflammatory biomarkers	1L-1*β*—promising candidate for differentiating AD from VD. Inflammatory pathways differ between dementias.
42	Maclin [57]	Systematic review	AD, VD, FD, LBD	Clinical setting	Biomarkers Neuroimaging	Neuroimaging with amyloid PET scanning surpasses what had been considered the dominant method of neuroimaging and MRI.
43	Malek-Ahmadi [58]	Meta-analysis	Amnestic MCI	General practice settings	Montreal Cognitive Assessment (MoCA)	MoCA: - AUC—0.84—good diagnostic accuracy
44	Martínez [59]	Systematic review	MCI, AD	Community, primary, secondary, and research centres	18F-florbetapir PET, amyloid PET imaging, NINCDS-ADRDA, DSM-IV dementia criteria.	Because of the poor sensitivity and specificity, the limited number of included participants, and the limited data available in the literature, 18F-florbetapir PET cannot be recommended for routine use in clinical practice to predict the progression from MCI to any form of dementia.
45	Martínez [60]	Systematic review	MCI, AD	Secondary care	18F PET with flutemetamol, NIA-AA criteria, NINCDS-ADRDA, and DSM-IV reference standards.	Evidence regarding 18F-flutemetamol PET accuracy in predicting progression from MCI to dementia remained limited and heterogeneous.
46	Mavroudis [61]	Meta-analysis	AD, PF, PSP, FTD, LBD	NA	CSF Alpha-synuclein Levels	CSF alpha-synuclein levels—different in LBD compared with AD, but no statistical difference was found between LBD and other dementias. Alpha-synuclein levels in the CSF can be used for the discrimination between LBD and AD.
47	Mavroudis [62]	Meta-analysis	MCI, AD, LBD	NA	CSF neurogranin levels	Neurogranin CSF levels - Higher in AD patients compared to MCI - No significant difference was found between AD and LBD - Higher levels in the CSF of MCI patients who progressed to AD, compared to stable MCI patients Neurogranin—could be added to the panel of existing biomarkers for a more accurate diagnosis of AD
48	McCarthy [63]	Systematic review	FTD	NA	Morphometric MRI	Good diagnostic accuracy—differentiating FTD from controls. Few machine learning algorithms have been tested in prospective replication. Studies are necessary before this method can be recommended for use clinically.
49	McCleery [64]	Systematic review	DLB	Secondary care	Dopamine transporter imaging	Only one study has used a neuropathological reference standard to assess the accuracy of DAT imaging for the diagnosis of DLB.
50	McCleery [65]	Systematic review	MCI, dementia	NA	Telehealth assessment	Telehealth assessment: - For the diagnosis of all-cause dementia, sensitivity ranged from 0.80 to 1.00, and specificity ranged from 0.80 to 1.00. For the diagnosis of MCI—one study—sensitivity of 0.71 and specificity of 0.73 Low-certainty evidence Tendency for patients identified as cognitively healthy at face-to-face assessment to be diagnosed with MCI at telehealth assessment (but numbers were small). Telehealth assessment may be sensitive and specific for the diagnosis of all-cause dementia when assessed against a reference standard of conventional face-to-face assessment. However, the estimates are imprecise due to small sample sizes and between-study heterogeneity. It may be applied mainly to telehealth models that incorporate a considerable face-to-face contact.
51	McGovern [66]	Systematic review	MCI and dementia in stroke	Various settings	Informant-Based Cognitive Screening IQCODE)	IQCODE: - For diagnosis of poststroke dementia—sensitivity of 0.81 and specificity of 0.83 Limited studies on informant cognitive assessments in stroke. IQCODE as a diagnostic tool
52	Micanovic [67]	Systematic review	Early-onset dementia (AD, VD, FTD, LBD)	NA	EEG	EEG abnormalities—more severe in early-onset AD patients, independent of disease duration. Slow wave activity is common to all dementias, but is most prominent in DLB. Frontal intermittent rhythmic delta activity—supportive for the diagnosis of DLB. EEG—usually normal in FTD. Focal changes—advanced VAD. EEG—useful as an adjunct in the diagnosis of DLB and AD.
53	Miller [68]	Systematic review	MCI, AD, VD, FTD, DLB, PDD	NA	Diagnostic criteria for apathy in NCD	Apathy evaluation—keeping the cognitive and behavioural domains separate for NCD without introducing a social withdrawal domain. Consensus diagnostic criteria for apathy in ND are provided
54	Namkoong [69]	Systematic review	AD	NA	Handgrip strength, pegboard hand dexterity tasks, and complex hand movement evaluations	Hand dexterity—associated with cognitive performance. Pegboard tasks—differentiated healthy older adults from AD patients. Complex hand movement analyses may help predict progression from MCI to AD and from moderate to severe dementia.
55	Nassanga [70]	Systematic review and meta-analysis	Dementia	Countries classified as low-income or middle-income	Brain MRI	The pooled prevalence of dementia-relevant MRI abnormalities was 58%, with substantial heterogeneity. Structured visual ratings may add aetiologic specificity beyond cognitive screening, but pooled estimates should be interpreted as summaries of heterogeneous studies.
56	Nihashi [71]	Systematic review and meta-analysis	DLB	Research setting	DAT-SPECT and MIBG scintigraphy	Both imaging biomarkers have high diagnostic accuracy for detecting the hallmark pathology in the brain and for diagnosing the typical clinical syndrome.
57	Noel-Storr [72]	Systematic review	Dementia	NA	Biomarkers b-amyloid, tau, positron emission tomography (18F-fluoro- deoxyglucose or ligands for amyloid), MRI	The highest number of studies—structural MRI The body of evidence for biomarkers is not large and is variable across the different types of biomarkers.
58	Ogonowski [73]	Systematic review	MCI, AD	Clinical and research setting	miRNA	Several peripheral fluid miRNAs showed potential as early biomarkers for MCI and AD-related cognitive impairment—further validation is needed.
59	Osei [74]	Systematic review	MCI, AD, FTD, DLB, PD	Resource-limited settings	NIRS	NIRS—effectively assesses cognitive function, identifying reduced prefrontal connectivity in MCI and subjective cognitive decline. NIRS—decreased oxyhemoglobin levels in AD patients’ dorsolateral cortex. Combining NIRS with graph analysis, cognitive tasks, and machine learning boosts diagnostic accuracy. NIRS can differentiate between neurodegenerative diseases. NIRS’s potential to improve cognitive assessment and neurodegeneration diagnosis.
60	Ossenkoppele [75]	Systematic review	AD	Specialized clinics for the differential diagnosis of dementia	Tau PET	Tau PET tracers: - Sensitive and specific for AD-related tau pathology and outperformed MRI markers diagnostically. - Promising tool for differential diagnosis between AD dementia and other neurodegenerative disorders in specialized clinics.
61	Ozer [76]	Systematic review	Amnestic MCI	Secondary care settings, community, mixture of secondary care and community-based settings	Brief cognitive screening tests	Several brief cognitive tests demonstrated promising sensitivity for identifying aMCI. Evidence quality and predictive validity remained limited.
62	Park [77]	Meta-analysis	MCI, AD	NA	MRI hippocampal volumetry and entorhinal cortex volumetry	Hippocampal volumetry: - For AD—sensitivity 82%, specificity 87% - For MCI—sensitivity 60%, specificity 75%. Entorhinal cortex volumetry demonstrated potentially superior performance for both AD and MCI. Entorhinal cortex volumetry: - For AD—sensitivity 88%, specificity 92% - For MCI—sensitivity 71%, specificity 83%
63	Pelegrini [78]	Systematic review	Dementia and cognitive dysfunction in the elderly	Primary healthcare, low-, middle-, and high-income countries	MCI and dementia criteria—based on experts’ recommendations and on the DSM and ICD-10 - most used instrument—MMSE	High-income countries—conducted by general practitioners—diagnostic criteria and instruments for assessments (cognitive and functional). Some used complementary evaluations—blood tests and neuroimaging. Middle-income countries—cognitive assessment
64	Pemberton [79]	Systematic review	Dementia	Research and clinical settings	Quantitative volumetric MRI reports tools	Automated volumetric MRI tools—potential to improve objectivity and consistency in dementia diagnosis. Clinical validation and workflow integration studies remain limited.
65	Piura [80]	Systematic review	Ultra-rapid progressive dementia	Tertiary care centres	CDR, clinical history and neurological examination, MRI, EEG, CSF analyses, disease-associated antibodies, FDG-PET, cognitive tests (MoCA, MMSE), diagnostic criteria for AD, bvFTD, DLB, CJD, autoimmune encephalitis, and vascular cognitive impairment	The evaluation of patients with ultra-RPD should prioritize testing for vascular, autoimmune/inflammatory, toxic/metabolic, and structural/iatrogenic causes, with priority given to the detection of potentially treatment-responsive causes of rapid dementia.
66	Qu [81]	Systematic review and meta-analysis	Amnestic MCI, AD	NA	Blood biomarkers (T-tau, P-tau, NfL, AβPPr, Aβ42, Aβ42/Aβ40 ratio, P-tau 217)	Biomarkers—valid in identifying AD and aMCI
67	Ramusino [82]	Updated systematic review	MCI, dementia	NA	Molecular imaging methods (FDG-PET, Amyloid-PET, DaT-SPECT, Tau-PET, MIBG scintigraphy)	FDG-PET and amyloid-PET—the most accurate molecular imaging biomarker in predicting MCI conversion to dementia. DaT-SPECT and MIBG—the most accurate biomarkers in distinguishing synucleinopathies and AD. FDG-PET—useful biomarker in the differential diagnosis between bvFTD and other dementias or psychiatric conditions, albeit with lower sensitivity and specificity. Use of biomarkers in ND other than AD—careful and comprehensive interpretation by expert clinicians
68	Ritchie [83]	Systematic review	MCI, AD	Several settings	Plasma and cerebrospinal fluid amyloid beta	Abnormally low CSF Aß levels—little diagnostic benefit with likelihood ratios suggesting only marginal clinical utility. We conclude that when applied to a population of patients with MCI, CSF Aß levels cannot be recommended as an accurate test for AD.
69	Ritchie [84]	Systematic review	MCI, AD	Clinical setting	CSF tau and the CSF tau/ABeta ratio	CSF tau and the CSF tau/ABeta ratio have better sensitivity than specificity. Insufficiency and heterogeneity of research—these tests may have limited clinical value until uncertainties have been addressed
70	Rizzo [85]	Systematic review and meta-analysis	LBD	Clinical setting	McKeith’s 1996 and 2005 criteria	Diagnostic criteria—become more sensitive and less specific over time, without a substantial change in the accuracy. About 20% of DLB diagnoses are incorrect.
71	Santos [86]	Systematic review	AD, FTLD, and DLB	Clinical and research settings	Blood biomarkers	Biomarkers for AD versus FTLD—excellent discriminative accuracy for p-tau181, p-tau217, synaptophysin, synaptopodin, GAP43, and calmodulin. For AD versus DLB distinction—excellent accuracy for miR-21-5p and miR-451a
72	Schäfer [87]	Systematic review	MCI, AD	NA	Word list learning tests	aMCI and early AD were associated with reduced item acquisition, more intrusion errors, reduced strategy use, and impaired recall. Process-based memory scores provided a more detailed characterization of episodic memory dysfunction than traditional total recall scores. Learning-process measures may improve early detection of AD-related memory decline.
73	Seitz [88]	Systematic review	AD	Primary care	Mini-Cog	Mini-Cog: Sensitivity—varied between 0.76 and 1.00 Specificity—varied between 0.27 and 0.85 There is a limited number of studies evaluating the accuracy of the Mini-Cog for the diagnosis of dementia in primary care settings. There is insufficient evidence to recommend Mini-Cog as a screening test for dementia in primary care.
74	Shahidi [89]	Systematic review and meta-analysis	MCI, AD	Clinical settings	MRI radiomics features analyzed with computational/machine learning models	MRI radiomics: - For differentiating AD from normal subjects—sensitivity 0.92 and specificity 0.91 - For differentiating MCI from normal subjects—sensitivity 0.74 and specificity 0.79 - For differentiating AD from MCI—sensitivity 0.73 and specificity 0.79
75	Shi [90]	Systematic review	Dementia	Clinical setting	Speech and language processing with deep learning	These technologies are promising in the diagnosis of dementia.
76	Smailagic [91]	Systematic review and meta-analysis	MCI, AD	Several settings	18F-FDG PET imaging	18F-FDG PET showed potential utility in predicting progression from MCI to AD dementia. Study heterogeneity and varying thresholds limited definitive conclusions regarding diagnostic accuracy.
77	Svensson [92]	Systematic review	Dementia	Community and primary healthcare settings—Asia and South America	Eight-item Informant Interview	Eight-item Informant Interview: - Sensitivity—88–100%, specificity—67–91% to differentiate ageing and dementia - Greater ability to predict people who are at risk of not having dementia than to correctly identify those at risk of having dementia
78	Tanwani [93]	Systematic review and meta-analysis	Dementia	Healthcare setting	AD8	Cut-off of >2/8 - Informant AD8 - Sensitivity of 80% and specificity of 79% to detect MCI. - Sensitivity of 91% and specificity of 64% to detect dementia. - Participant AD8, - Sensitivity of 57% and specificity of 71% to detect MCI - Sensitivity of 82% and specificity of 75% to detect dementia. The AD8 may be an acceptable alternative to screen for cognitive impairment in older adults when there are limitations to formal testing.
79	Tseriotis [94]	Systematic review and meta-analysis	LBD	NA	Loss of DNH on iron-sensitive brain MRI	DNH loss on iron-sensitive MRI—might comprise a supportive biomarker for DLB detection
80	Verón [95]	Systematic review	MCI, AD, and related dementias	NA	Eye tracking	Antisaccade tasks consistently distinguished AD and MCI from healthy controls.
81	Wang [96]	Meta-analysis	MCI due to AD	NA	MMSE, MoCA	MoCA—superior diagnostic performance compared with MMSE for identifying MCI due to AD, showing higher sensitivity, specificity, and diagnostic odds ratio. MoCA—a more effective screening tool for early cognitive impairment.
82	Wang [97]	Meta-analysis	MCI, AD	Clinical setting	MRI-based deep learning diagnosis	Deep learning of MRI for the diagnosis of AD and MCI—good sensitivity and specificity
83	Weissberger [98]	Systematic review and meta-analysis	MCI, AD	Clinical setting	Neuropsychological memory measures—immediate and delayed verbal/visual recall, associative learning tasks, recognition memory tests	Neuropsychological measures of memory—valid cognitive biomarkers of AD.
84	Xie [99]	Meta-analysis	MCI, AD	Clinical setting	Peripheral BDNF levels	Deregulation of BDNF—a possible contributor to the pathology and symptoms of AD. AD or MCI—reduction in peripheral BDNF. ROC curve analysis—peripheral BDNF levels may not be an optimal biomarker for AD and MCI diagnosis.
85	Xing [100]	Meta-analysis	MCI, AD	NA	Exosome-derived biomarker	Exosome-derived biomarkers—high diagnostic value for AD and MCI. Sample type, type of exosomal content, and sample size impacted the biomarkers’ diagnostic value in AD and MCI. Present results could not distinguish between different stages of AD and MCI based solely on biomarker expression levels.
86	Yeo [101]	Systematic review	AD, FTD, VD	NA	SPECT - ^99m^Tc-hexamethylpropyleneamine (^99m^Tc-HMPAO) ^99m^Tc-ethylcysteine dimer	Pooled weighted sensitivity and specificity of ^99m^Tc-HMPAO-SPECT in distinguishing: - Clinically diagnosed AD from FTD—79.7 and 79.9%, respectively - AD from VD—74.5 and 72.4% - AD from DLB—70.2 and 76.2% - AD from NC—76.1 and 85.4%. SPECT—diagnostic value particularly in differentiating Alzheimer’s disease from frontotemporal dementia and normal controls—should not be used in isolation but rather as an adjunct and interpreted in the context of clinical information and paraclinical test results.
87	Zhang [102]	Systematic review and meta-analysis	MCI, AD	Several settings (secondary, tertiary, mixed)	11C-PIB-PET, NINCDS-ADRDA, DSM-IV criteria	PIB-PET showed high sensitivity for predicting progression from MCI to AD dementia, but specificity was limited, and study heterogeneity was substantial. PIB-PET—should not yet be routinely recommended in clinical practice.
88	Zhu [103]	Systematic review and meta-analysis	MCI	NA	FDG-PET, SPECT, and MRI imaging	The sensitivity and specificity of FDG-PET imaging were significantly higher than those of SPECT and MRI imaging. Cerebral perfusion imaging—good prognostic value for patients with MCI. FDG-PET imaging—better predictive ability of the prognosis for patients with MCI.

*Note.* ACE-R Addenbrooke’s Cognitive Examination-Revised; AD = Alzheimer’s disease; AD8 = Ascertain Dementia 8; AMTS = Abbreviated Mental Test Score; ASL-MRI = Arterial spin labelling; ASR = automatic speech recognition; BDNF = brain-derived neurotrophic factor; CAM = confusion assessment method; CJD = Creutzfeldt–Jakob disease; DAT = dopamine transporter; DNH = dorsolateral nigral hyperintensity; DSM = Diagnostic and Statistical Manual of Mental Disorders; EFNS = European Federation of Neurological Societies; ERP = event-related potential; FDG-PET = fluorodeoxyglucose-PET; FTD = frontotemporal dementia; FTLD = frontotemporal lobar degeneration; GPCOG = General Practitioner Assessment of Cognition; HD = Huntington’s disease; ICD = International Classification of Diseases; IQCODE = Informant Questionnaire on Cognitive Decline in the Elderly; LBD = Lewy body dementia; MCI = Mild Cognitive Impairment; ML = Machine Learning; MIBG = I-Meta-iodobenzyl-guanidine; MIS = Memory Impairment Screen; MMSE = Mini-Mental State Examination; MoCA = Montreal Cognitive Assessment; ND = Neurocognitive disorder; NFL = neurofilament light chain; NIA-AA = National Institute on Ageing/Alzheimer’s Association; NINCDS-ADRDA = National Institute of Neurological and Communicative Disorders and Stroke and the Alzheimer’s Disease and Related Disorders Association; NIRS = Near-infrared spectroscopy; NLP = natural language processing; PET = positron emission tomography; PSP = progressive supranuclear palsy; QMCi = Quick Mild Cognitive Impairment Screen; TOM = theory of mind; TYM = Test Your Memory; VCAT = visual cognitive assessment test; VCI = vascular cognitive impairment; VD = vascular dementia; 4AT = 4-A’s Test; 6CIT = 6-item cognitive impairment test; 6-IS = 6-item screener; 10-CS = 10-point cognitive screener.

**Table 3:** Summary of guidelines for translational research and clinical application of the MOND Model.

Phase	Translational Step	Objective and Description	Expected Practical Output
**I. Assessment formulation**	**1. Tool identification**	Select and validate specific instruments to assess MOND factors (e.g., Addenbrooke’s Cognitive Examination-III (ACE-III) for Neurocognition; Neuropsychiatric Inventory (NPI) for Neuropsychiatric Symptoms; **Unified Parkinson’s Disease Rating Scale** (UPDRS)/Kinematics for Motor Symptoms; Edinburgh Social Cognition Test (ESCoT) for Social Neurocognition; Inventory of sensory, emotional, and cognitive reserve (SECRI) for Cognitive, Emotional, and Sensory Reserve; Activities of Daily Living Inventory (ADLI) for Functionality).	Standardized multidimensional assessment battery.
	**2. Multidisciplinary interview**	Create a collaborative, semi-structured clinical interview framed around the MOND model (e.g., integrating neurology and neuropsychology).	Standardized clinical interview protocol.
**II. Empirical validation**	**3. Preliminary testing**	Apply the framework to participants to identify qualitative and quantitative patterns of functioning across ND severities and healthy ageing.	Baseline clinical dataset and pattern identification.
	**4. Interaction analyses**	Conduct mediation and moderation analyses to understand how factors (e.g., reserve, comorbidities) interact and influence ND progression.	Statistical interaction models and precise ND risk estimation algorithms.
**III. Clinical integration**	**5. Model refinement**	Iteratively adjust and refine the theoretical framework based on the empirical results of the moderation and mediation analyses.	Updated, evidence-based MOND framework.
	**6. Clinical pathways**	Define structured decision trees to guide diagnosis and prognosis based on the patient’s unique multidimensional profile.	Personalized prognoses, tailored treatment strategies, and clinical decision trees.

## Data Availability

No new data were created or analyzed in this study.
